# On the Contagiousness of Hooping-Cough

**Published:** 1856-04

**Authors:** 


*On the contagiousness of Hooping-cough.* By Professor Trousseau.

(Medical Times and Gazette, June 2, 1855.)

The following remarks occur in a clinical lecture recently delivered in the Hotel Dieu at Paris:

"We have had a slight epidemic of pertussis in the children's wards, where it was introduced by a single case. During a certain period, all the children in the ward, and those who were admitted, acquired the disease. Then, at a certain period, although there were from eight to ten children at the height of the disease, others were admitted without any of them catching it. Thus to render contagion possible, certain conditions are necessary, which are no less real for being undetermined."—*Ranking's Abstract.*

